# Quantification of the neurochemical profile of the human putamen using STEAM MRS in a cohort of elderly subjects at 3 T and 7 T: Ruminations on the correction strategy for the tissue voxel composition

**DOI:** 10.1371/journal.pone.0286633

**Published:** 2023-06-02

**Authors:** Ana Gogishvili, Ezequiel Farrher, Christopher E. J. Doppler, Aline Seger, Michael Sommerauer, N. Jon Shah

**Affiliations:** 1 Institute of Neuroscience and Medicine 4, INM-4, Forschungszentrum Jülich, Jülich, Germany; 2 Faculty of Medicine, RWTH Aachen University, Aachen, Germany; 3 Engineering Physics Department, Georgian Technical University, Tbilisi, Georgia; 4 Cognitive Neuroscience, Institute of Neuroscience and Medicine 3, INM-3, Forschungszentrum Jülich, Jülich, Germany; 5 Department of Neurology, Faculty of Medicine and University Hospital Cologne, University of Cologne, Cologne, Germany; 6 Institute of Neuroscience and Medicine 11, INM-11, JARA, Forschungszentrum Jülich, Jülich, Germany; 7 JARA – BRAIN - Translational Medicine, Aachen, Germany; 8 Department of Neurology, RWTH Aachen University, Aachen, Germany; Museo Storico della Fisica e Centro Studi e Ricerche Enrico Fermi, ITALY

## Abstract

The aim of this work is to quantify the metabolic profile of the human putamen *in vivo* in a cohort of elderly subjects using single-voxel proton magnetic resonance spectroscopy. To obtain metabolite concentrations specific to the putamen, we investigated a correction method previously proposed to account for the tissue composition of the volume of interest. We compared the method with the conventional approach, which *a priori* assumes equal metabolite concentrations in GM and WM. Finally, we compared the concentrations acquired at 3 Tesla (T) and 7 T MRI scanners. Spectra were acquired from 15 subjects (age: 67.7 ± 8.3 years) at 3 T and 7 T, using an ultra-short echo time, stimulated echo acquisition mode sequence. To robustly estimate the WM-to-GM metabolite concentration ratio, five additional subjects were measured for whom the MRS voxel was deliberately shifted from the putamen in order to increase the covered amount of surrounding WM. The concentration and WM-to-GM concentration ratio for 16 metabolites were reliably estimated. These ratios ranged from ~0.3 for γ-aminobutyric acid to ~4 for *N*-acetylaspartylglutamate. The investigated correction method led to significant changes in concentrations compared to the conventional method, provided that the ratio significantly differed from unity. Finally, we demonstrated that differences in tissue voxel composition cannot fully account for the observed concentration difference between field strengths. We provide not only a fully comprehensive quantification of the neurochemical profile of the putamen in elderly subjects, but also a quantification of the WM-to-GM concentration ratio. This knowledge may serve as a basis for future studies with varying tissue voxel composition, either due to tissue atrophy, inconsistent voxel positioning or simply when pooling data from different voxel locations.

## 1. Introduction

Proton magnetic resonance spectroscopy (1H-MRS) enables the quantification of metabolite concentrations *in vivo*. It has proven to be a valuable tool for the assessment of several pathologies and disorders of the human brain, such as tumours [1], Alzheimer’s disease [2, 3] and Parkinson’s disease [4, 5], as well as for the study of healthy ageing [6, 7]. Although the added value of 1H-MRS is greatly enhanced if the metabolite concentration is provided in absolute units (e.g. moles per kilogram or litre of tissue), the majority of the clinical studies still make use of either relative concentrations (to total creatine (tCr) or total *N*-acetylaspartate (tNAA)) [8] or absolute concentrations without appropriate corrections (conventionally expressed in institutional units, [i.u.]) [9]. However, as concentrations of tCr and tNAA have been shown to change in certain brain pathologies [10] or even between brain regions [11], the applicability of this approach is clearly limited. The use of institutional units, on the other hand, restricts the use of the quantified concentrations to within studies, making it difficult to reproduce and compare results.

To achieve absolute quantification, several factors must be considered, including correction for signal relaxation and the use of a signal reference. The use of the unsuppressed tissue water signal as an internal reference has been shown to be of great value in quantifying the metabolic profile of the brain *in vivo* [12–15] since it enables the omission of certain correction factors, e.g. the radiofrequency (RF) field inhomogeneity. Techniques to account for the differences in tissue water concentration are widely used in research [8, 13, 16, 17]. Conversely, although the difference in metabolite concentration between white (WM) and grey (GM) matter has already been studied [18], the correction for such difference in the context of quantitative single-voxel MRS has received only minor attention [18–20]. This issue is particularly crucial for studies that include patients with brain atrophy [21] or in longitudinal studies where the positioning of the volume of interest (VOI) is not consistent [22]. Moreover, given the ubiquity of the partial volume effect (PVE) in single-voxel MRS, metabolite concentration ratios with the surrounding tissue also need to be accounted for when quantifying the metabolic profile of a specific brain structure. A possible mitigation of the problem with PVE in single-voxel MRS is to use the VOI tissue fractions as a covariate in the statistical analysis [21, 22]. However, a limitation of this approach is that the absolute metabolite concentration of the structure of interest remains unknown. An alternative approach was proposed by Harris et al. [19], in which, in addition to the conventional tissue-specific water concentration and signal relaxation, the WM-to-GM metabolite concentration ratio (termed from now on as the *α*_m_-ratio) is explicitly considered. In particular, Harris et al. applied the method for quantifying the γ-aminobutyric acid (GABA) concentration in several brain areas, assuming a ratio of 0.5 (i.e., there is twice as much GABA in GM compared to WM). This approach was later shown to have a significant impact on the statistical analysis of GABA concentrations in healthy ageing: a significant dependency of GABA on age was observed if no correction was used, whereas the dependency was negligible if the *α*_m_-ratio was considered [22]. Nonetheless, to the best of our knowledge, the method has not been applied to investigate other metabolites.

The primary goal of this study is to quantify the neurochemical profile of the human putamen *in vivo* using pre-existing experimental spectra from healthy control subjects. The data were acquired in a case-control study on Parkinson’s disease using an ultra-short TE, single-voxel stimulated echo acquisition mode (STEAM) [23, 24] sequence at 3 Tesla (T) and 7 T [4]. The putamen was chosen due to its central role in the pathophysiology of Parkinson’s disease. Besides commonly applied corrections, such as transverse (*T*_2_) and longitudinal (*T*_1_) relaxation effects [25], we also considered the correction for the *α*_m_-ratio [19]. This was achieved by first assessing the metabolite concentration *α*_m_-ratio following the method proposed by Hetherington et al. [18]. The effect of incorporating the *α*_m_-ratio into the correction method was then compared to the case where that feature was neglected. Finally, we compared the quantified neurochemical profiles achieved at 3 T and 7 T field strengths as a means of *in vivo* validation [25].

## 2. Materials and methods

### 2.1 Quantification methods

Both of the quantification methods we utilised make use of the unsuppressed water signal acquired from the same MRS voxel as the internal reference [13] and the concentration output from the software LCModel [26]. The conventional correction method (M_1_) considers the metabolite concentration in CSF to be negligible compared to that of brain tissue. It takes into account that water concentration changes according to the tissue type [13, 27–29] and contains factors accounting for *T*_1_ and *T*_2_ relaxation effects in both the metabolite and water reference signals. The metabolite concentration in this case is written as follows [13, 25]:

$$c_{m,1}=A\;\frac{\sum_{i}c_{w,i}E_{w,i}^{\left(1\right)}E_{w,i}^{\left(2\right)}f_{i}}{\;E_{m}^{\left(1\right)}E_{m}^{\left(2\right)}{(f}_{GM}+f_{WM})}$$ (1)

where *A* is the metabolite concentration from LCModel [i.u.] (assuming the default relaxation and the water content correction factors in LCModel, ATTH2O and WCONC, equal 1), ƒ_*i*_ are the tissue volume fractions within the VOI, and *c*_w,*i*_ are the tissue water concentrations (*i* = WM, GM and CSF). Here we assume *c*_w,WM_ = 36100 mM, *c*_w,GM_ = 43300 mM, and *c*_w,CSF_ = 53800 mM [13]. *E*^(1)^ and *E*^(2)^ are the *T*_1_ and *T*_2_ relaxation attenuation factors which, for the STEAM sequence, are [30]:

$$E^{\left(1\right)}=\left\{1-exp\left[\left(\frac{TE}{2}+TM-TR\right)\frac{1}{T_{1}}\right]\right\}exp\left(-\frac{TM}{T_{1}}\right)$$

and

$$E^{\left(2\right)}=exp\left(-\frac{TE}{T_{2}}\right),$$

where TM is the mixing time. *E*^(1)^ and *E*^(2)^ corrections can only be neglected when TR ≫ *T*_1_, TM ≪ *T*_1_ and TE ≪ *T*_2_. Note that both relaxation terms for the metabolite case are assumed to be the same for WM and GM [13, 31, 32].

The second method (M_2_) utilises the same corrections as in M_1_, but with the additional consideration that metabolite concentrations in WM and GM may be different. The metabolite concentration can be written as [19]:

$$c_{m,2}=A\;\frac{\sum_{i}c_{w,i}E_{w,i}^{\left(1\right)}E_{w,i}^{\left(2\right)}f_{i}}{E_{m}^{\left(1\right)}E_{m}^{\left(2\right)}{(f}_{GM}+{\alpha_{m}f}_{WM})},$$ (2)

where *α*_m_ = *c*_WM_/*c*_GM_ is the WM-to-GM metabolite concentration ratio, and *c*_WM_ and *c*_GM_ are metabolite concentrations for pure WM and GM, respectively. Notice that *c*_m,2_ in Eq (2) represents what the measured concentration would be if the VOI were entirely filled by GM [19].

### 2.2 Estimation of the concentration ratio α_m_

The pure WM and GM metabolite concentrations, and consequently the concentration ratio, *α*_m_, can be assessed by fitting a linear function to the concentration calculated with M_1_ vs. the normalised GM volume fraction, *φ*_GM_ = ƒ_GM_/(ƒ_GM_+ƒ_WM_) [19]. The linear function can be expressed as

$$c_{m,1}\left(\varphi_{GM}\right)=\left(c_{GM}-\;c_{WM}\right)\varphi_{GM}+c_{WM},$$ (3)

where *φ*_GM_ lies in the range [0,1] and *c*_GM_ and *c*_WM_ are free parameters. Clearly, a sufficiently broad *φ*_GM_ range needs to be covered to achieve a robust estimation of *c*_GM_ and *c*_WM_. However, most studies seek to position the VOI in a consistent manner throughout the subjects, inevitably leading to a rather small *φ*_GM_ range. Consequently, some works have opted to pool the data acquired from different voxel locations to achieve a more robust estimation of *α*_m_ [19, 33].

In order to consider the effect of a potential bias in the water relaxation times or tissue water content used in Eqs (1) and (2) in *α*_m_, we evaluated the relative change, Δ*α*_m_, as follows

$${\Delta \alpha }_{m}=\frac{\alpha_{m}(b)-\alpha_{m}(b_{0})}{\alpha_{m\;}(b_{0})}\times 100\%,$$ (4)

where the parameter *b* can be either *T*_1_, *T*_2_, *c*_w,WM_ or *c*_w,GM_ and the subscript “0” denotes the unbiased counterpart. For the sake of simplicity, an equal bias in the water relaxation times for both WM and GM was assumed. Notice that due to the intrinsic assumption of non-distinct metabolite relaxation times between WM and GM in Eq (1), a bias in the metabolite relaxation times will not affect Δ*α*_m_.

Finally, to investigate the way in which a possible bias in the estimated concentration ratio propagates into the metabolite concentration obtained following M_2_, we defined the error, *ε*, in the absolute concentration if the concentration ratio used, i.e. *α*_m_, differs from the actual concentration ratio, *α*_m,0_, as follows:

$$\varepsilon =\frac{c_{m,2}(\alpha_{m})-c_{m,2}(\alpha_{m,0})}{c_{m,2\;}(\alpha_{m,0})}\times 100\%.$$ (5)

### 2.3 Subjects

MRS spectra were acquired from the putamen of 15 healthy subjects (age 53 to 80 years, mean 67.7 ± 8.3 years, six females and nine males) in the framework of a case-control Parkinson’s disease study [4]. Due to the limited range of *φ*_GM_ in the MRS voxels of that study, five more age-matched subjects (three VOIs each) were measured. For these subjects, the VOI was deliberately shifted to include higher fractions of WM (see Section 2.4 *MR protocol*). The Parkinson’s disease study was approved by the Ethics Committee of the Medical Faculty of the University of Cologne, Germany. The five extra subjects were measured under the approval of the Ethics Committee of the Medical Faculty of the RWTH Aachen University, Germany. All participants gave written informed consent according to the Declaration of Helsinki.

### 2.4 MR protocol

For all subjects, spectra were initially acquired on a Siemens 3 T hybrid PET/MR Tim Trio scanner (Siemens Medical Solutions, Erlangen, Germany) and then on a Siemens 7 T Terra scanner. The 3 T scanner was equipped with a birdcage transmit coil and an 8-channel receive coil provided by the vendor, whereas the 7 T scanner used a single transmit coil with a 32-channel receive coil (Nova Medical, USA). The MPRAGE sequence [34] was used to position the VOIs in the putamen at 3 T. The protocol parameters were: TR = 2.5 s; TE = 2.89 ms; voxel-size = 1 × 1 × 1 mm^3^; matrix-size = 176 × 232 × 256; flip angle, 7º. At 7 T, the MP2RAGE sequence [35] was utilised for the same purpose. The protocol parameters were: TR = 4.5 s; TE = 1.99 ms; voxel-size = 0.75 × 0.75 × 0.75 mm^3^; matrix-size = 208 × 300 × 320; flip angle, 5º.

First- and second-order B0 shimming at the VOI was performed using FASTESTMAP [36], and the RF power was calibrated for each subject [37, 38]. Water suppression was achieved with VAPOR and was interleaved with outer volume suppression (OVS) [23, 39]. Spectra were measured using the STEAM sequence [6, 23], with the voxel centred in the left putamen. The protocol parameters at 3 T were: TE / TR / TM = 6 / 4800 / 47.8 ms; 128 averages; voxel-size = 21 (left-right) × 35 (anterior-posterior) × 21 (rostral-caudal) mm^3^; receive-bandwidth, 2000 Hz; vector-size = 2048; flip angle = 90°; RF pulse duration = 1920 μs. At 7 T, the protocol parameters were: TE / TR / TM = 4 / 8000 / 28 ms, 72 averages; voxel-size = 14 (left-right) × 32 (anterior-posterior) × 17 (rostral-caudal) mm^3^; receive-bandwidth, 6000 Hz; vector-size = 2048; flip angle = 90°; RF pulse duration = 1920 μs. One extra complete phase cycle was measured without the water suppression RF pulses for eddy-current correction and absolute quantification. The same 3 T and 7 T MRS protocols were also used for the five additional subjects, for whom three different voxel positions were measured, namely occipital (WM_1_), parietal (WM_2_), and frontal (WM_3_) WM (S1 Fig).

### 2.5 Data preprocessing and quantification

All data sets from the 3 T and 7 T scanners were processed following the same pipeline with the help of the FID-A package [40], available for Matlab (Natick, MA, USA). The preprocessing steps included i) automatic detection and removal of motion corrupted scans [40] and ii) phase and frequency drift correction of individual averages using spectral registration in the frequency domain [41]. Analysis of the preprocessed data was performed using LCModel (6.3-1R) with the water scaling and eddy-current correction options enabled. Fitting was performed in the chemical shift range of 0.2 ppm to 4.2 ppm. The metabolite basis sets were generated with the help of the tool VeSPA [42] using the density matrix formalism [43] with ideal RF pulses and actual sequence timings [44, 45]. Both basis sets included spectra of 19 metabolites: alanine (Ala), ascorbate (Asc), aspartate (Asp), creatine (Cr), GABA, glucose (Glc), glutamine (Gln), glutamate (Glu), glutathione (GSH), glycerophosphorylcholine (GPC), *myo*-inositol (Ins), lactate (Lac), NAA, *N*-acetylaspartylglutamate (NAAG), phosphocreatine (PCr), phosphorylcholine (PCh), phosphorylethanolamine (PE), *scyllo*-inositol (Scyllo), taurine (Tau). Macromolecular spectra measured using the inversion recovery STEAM sequence at 3 T (TE = 8 ms) and 7 T (TE = 6 ms) available at MM Consensus Data Collection (https://github.com/mrshub/mm-consensus-data-collection), were additionally used in the basis sets [24, 46]. The default rigidity parameter was used for the baseline in LCModel (DKNTMN = 0.15). A metabolite was considered to be reliably estimated if measured in at least 50% of the subjects with a Cramér-Rao lower bound (CRLB) value less or equal to 50% [47, 48]. Only spectra with the LCModel full width at half maximum output (FWHM) lower than 0.07 ppm were considered. Moreover, if the correlation coefficient between two metabolites was less than -0.7, only their sum was reported (e.g. tCho [GPC+PCh] and tCr [Cr+PCr]), whereas both the total concentration and the individual concentrations (e.g. tNAA [NAA+NAAG]) were reported for metabolites showing a correlation of between -0.7 and -0.3 [47, 48].

### 2.6 Tissue segmentation

An important issue in metabolite quantification, as well as in the estimation of the *α*_m_-ratio, is the tissue segmentation approach [19]. In contrast to cortical segmentation, which assumes the same structural features for the whole cortex, segmentation of subcortical structures requires methods that are specific to these structures [49]. Traditional methods, such as FAST [50], face problems with the segmentation of subcortical GM in single contrast images, e.g. the *T*_1_-weighted images in this work, due to its limited contrast with WM. Therefore, given that the putamen VOI in our study contains both cortical and subcortical GM, in order to assess the tissue volume fractions, we utilised a combined approach in which FAST was used for the segmentation of cortical GM, WM, and CSF, and FIRST [51] was used for subcortical GM. Both tools are available as part of FSL (FMRIB Software Library v6.0.3).

### 2.7 Statistical analysis

Prior to the estimation of the *α*_m_-ratio, the distributions of metabolite concentrations evaluated using M_1_ (*c*_m,1_) were compared between the scanners. If *c*_m,1_ concentrations at 3 T vs. 7 T were not significantly different, then the pooled *c*_m,1_ concentrations (3 T and 7 T) were used for the estimation of the *α*_m_-ratio. Otherwise, 3 T and 7 T *c*_m,1_ were considered separately, and the *α*_m_-ratio was estimated for each field strength. A one‐way analysis of covariance (ANCOVA) was used to compare the *c*_m,1_ concentrations between the scanners, where *φ*_GM_ at each scanner was used as the covariate. This was performed with the help of the SPSS software (28.0.1.0 (142)).

The metabolite concentrations obtained using the correction methods M_1_ and M_2_ were compared for each field strength separately. To this end, a two-sample t-test was performed for each group of subjects. Additionally, a scanner comparison was performed for each of the correction methods utilising a two-sample t-test.

A further evaluation of the effect of using the *α*_m_-ratio method (M_2_) compared to the case of neglecting it (M_1_) was performed by calculating the Pearson’s correlation coefficient of the concentrations *c*_m,1_ (and *c*_m,2_) vs. *φ*_GM_. While a significant correlation of *c*_m,1_ vs. *φ*_GM_ for a given metabolite denotes that *α*_m_ ≠ 1 (i.e. different WM and GM concentrations), a lack of a correlation of *c*_m,2_ vs. *φ*_GM_ for the same metabolite signifies that the use of method M_2_ can indeed remove the dependence of the absolute metabolite concentration on the tissue voxel composition.

## 3. Results

### 3.1 Tissue segmentation

**Fig 1:**
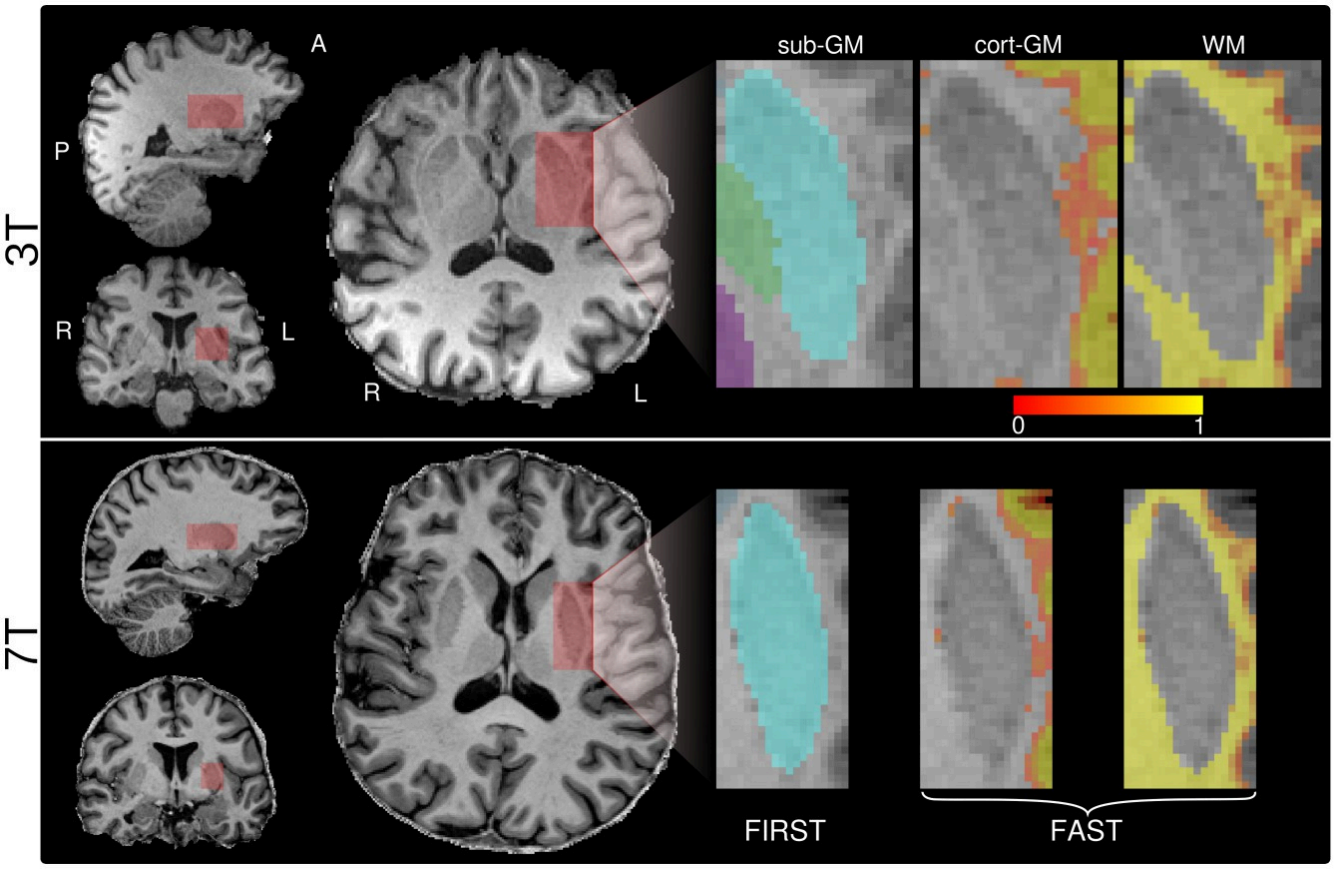
Examples of the putamen VOI positioning at 3 T (top panel) and 7 T (bottom panel) overlaid to the respective anatomical images. Tissue segmentation using FIRST for subcortical structures and FAST for cortical GM and WM are depicted. In the case of subcortical GM, the colours refer to: light blue, putamen (PUT); green, globus pallidus; violet, thalamus. In the case of FAST, the colour scale refers to the cortical GM/WM volume fraction.

Fig 1 depicts the MRS voxel position and corresponding tissue segmentation for a representative subject from the putamen group measured at 3 T (top row) and 7 T (bottom row). The volume fractions averaged across subjects are shown in Table 1. Likewise, the fraction of putamen covered by the MRS voxel was 0.89 ± 0.03 (3 T) and 0.76 ± 0.04 (7 T). The corresponding VOI position of the WM voxels is shown in S1 Fig.

**Table 1:** Mean and standard deviation of the VOI tissue composition across subjects.

	WM	GM	CSF
PUT 3 T	0.37 ± 0.02	0.60 ± 0.03	0.03 ± 0.01
PUT 7 T	0.34 ± 0.06	0.66 ± 0.06	0.003 ± 0.002
WM_1_ 3 T	0.87 ± 0.04	0.10 ± 0.04	0.03 ± 0.01
WM_1_ 7 T	0.95 ± 0.03	0.04 ± 0.02	0.01 ± 0.01
WM_2_ 3 T	0.62 ± 0.09	0.35 ± 0.11	0.03 ± 0.01
WM_2_ 7 T	0.74 ± 0.13	0.25 ± 0.12	0.01 ± 0.12
WM_3_ 3 T	0.82 ± 0.07	0.13 ± 0.05	0.05 ± 0.02
WM_3_ 7 T	0.88 ± 0.06	0.10 ± 0.04	0.02 ± 0.02

### 3.2 Water linewidth and spectral quality

**Fig 2:**
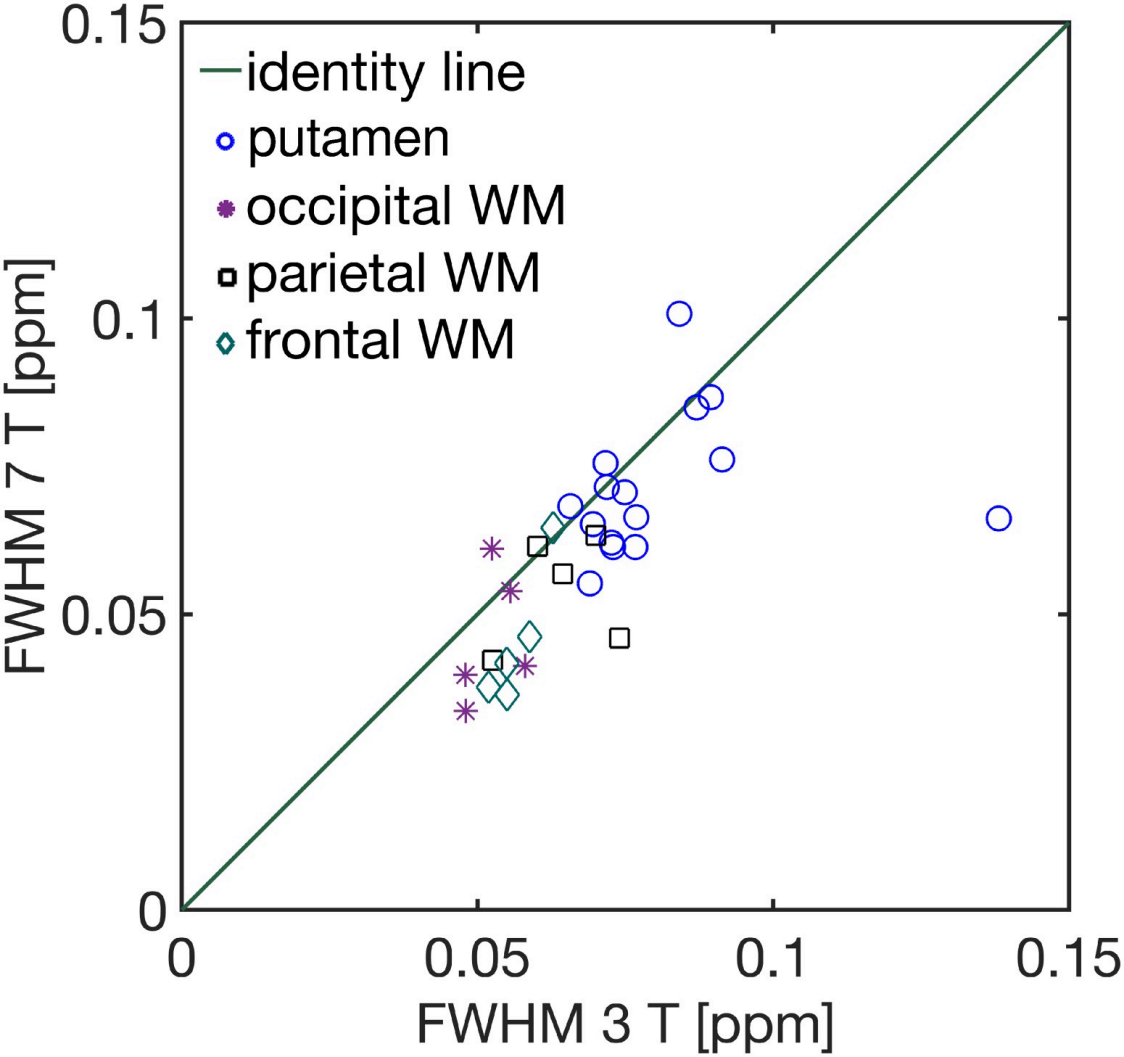
Linewidth of the unsuppressed water signal for the individual subjects achieved at 3 T vs. 7 T. ∘: putamen; *: occipital WM; ⧠: parietal WM; ◊: frontal WM. Note that the subject with linewidth 0.14 ppm was excluded from the correlation due to poor B0 shimming.

Fig 2 shows the correlation between the linewidth of the unsuppressed water signals (i.e. FWHM of the water peak estimated with the help of the FID-A package [40]) acquired at both field strengths. The Pearson’s correlation coefficient of the linewidth values at 3 T vs. 7 T was *r* = 0.83; p < 10^−7^. The mean linewidth values for the putamen, averaged across subjects, were 0.077 ± 0.008 ppm (3 T) and 0.07 ± 0.01 ppm (7 T), 0.052 ± 0.005 ppm (3 T) and 0.05 ± 0.01 ppm (7 T) for occipital WM, 0.064 ± 0.008 ppm (3 T) and 0.054 ± 0.009 ppm (7 T) for parietal WM, and 0.057 ± 0.004 ppm (3 T) and 0.05 ± 0.01 ppm (7 T) for frontal WM.

**Fig 3:**
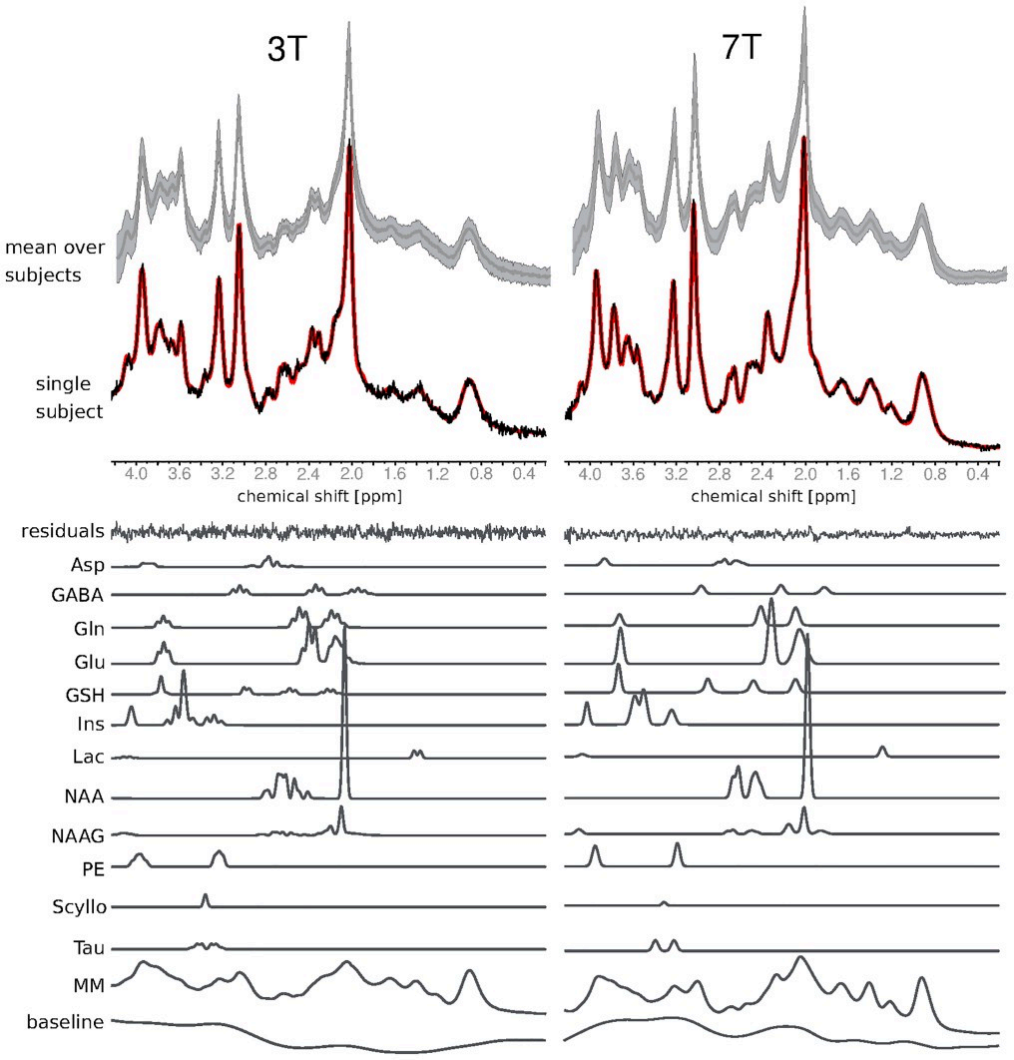
Top: mean (grey, solid line) and standard deviation (grey, shaded area) of the experimental spectra obtained from the putamen at 3 T (left) and 7 T (right). The spectra of a representative subject (black, solid line) are shown overlaid to the corresponding LCModel fit (red, solid line) together with the corresponding residuals of the fit. The individual metabolite contribution to the total spectra obtained with LCModel is additionally shown at the bottom of the figure.

A mean of 6.9 averages (5.4% of the number of averages) were discarded from the total number of averages at 3 T, whereas at 7 T, a mean of 2.4 (3.3%) averages were discarded. The spectral characteristics of the putamen VOI at 3 T (left column) and 7 T (right column) are depicted in Fig 3. The mean (grey, solid line) and standard deviation (grey, shaded area) of the measured spectra taken over the subjects in the putamen group are shown at the top of the figure. Additionally, the individual spectra for both scanners are shown in S2 Fig. Single spectra (black, solid lines), together with the corresponding LCModel fit (red, solid line), the residuals, and the individual metabolite contributions are additionally shown for a representative subject. The random distributions of the residuals indicate a good fitting of the spectra achieved with LCModel at both field strengths. A total of 12 metabolites and four total concentrations (tCho, tNAA, tCr, and Glx (Gln+Glu)) were reliably measured following the conditions described in Section 2.5. In the case of the pairs Cr-PCr and GPC-PCh, the correlation was < -0.7 for both field strengths, whereas for the pair NAA-NAAG the mean correlation was equal to -0.65 (3 T) and -0.49 (7 T). Scyllo was only reliably detected at 3 T. The average signal-to-noise ratio (SNR) values obtained with LCModel (defined as the ratio of the maximum in the spectrum minus the baseline to twice the root-mean-square of the residuals) were 31 ± 3 at 3 T and 34 ± 9 at 7 T. The metabolite concentrations as outputted by LCModel without further corrections averaged over the whole putamen group for both scanners are indicated in S1 Table.

### 3.3 Concentration ratio α_m_

**Fig 4:**
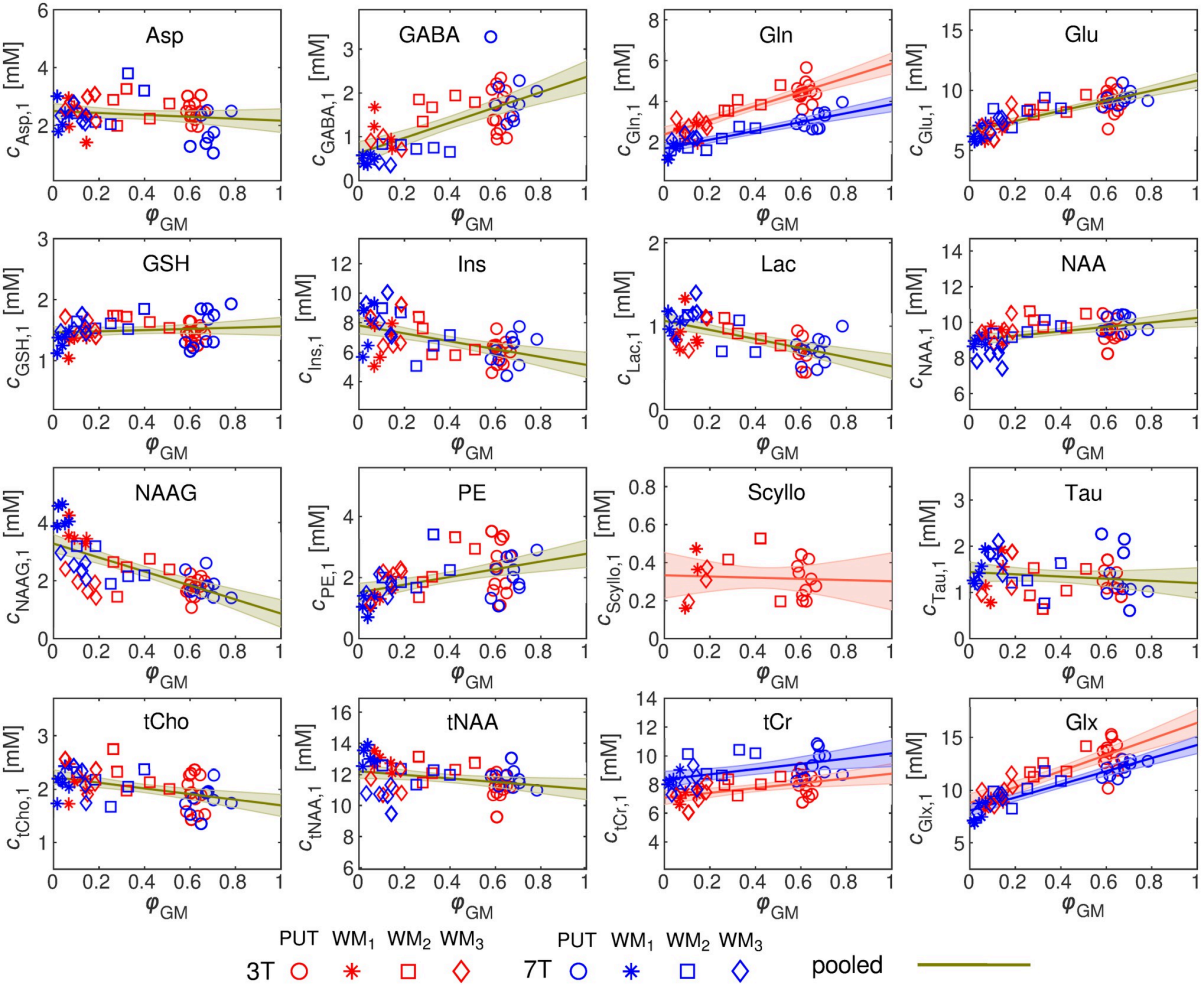
Plots of the *c*_m,1_ concentrations vs. *φ*_GM_ for 3 T (red) and 7 T (blue) data, and for the whole metabolic profile. The regression lines (solid lines, Eq (3)), together with the 95% confidence bands (shaded areas), are additionally shown. Note that Gln, tCr and Glx distributions were significantly different between the scanners, as assessed by the ANCOVA analysis using *φ*_GM_ as the covariate (p < 0.05). Therefore, the linear regression was performed separately for 3 T (red line) and 7 T (blue line) for these metabolites. For the rest of the metabolites, the linear regression (green lines) was performed using the pooled data. All brain areas were considered for the linear regression. o: putamen (PUT); *: occipital WM; ⧠: parietal WM; ◊: frontal WM.

Fig 4 shows the metabolite concentrations quantified using method M_1_ (Eq (1)), *c*_m,1_, plotted against the normalised volume fraction, *φ*_GM_. The water and metabolite relaxation times used in Eqs (1) and (2) are indicated in S2 Table. The ANCOVA analysis using *φ*_GM_ as the covariate showed that the *c*_m,1_ distributions of Gln, tCr, and Glx were significantly different (p < 0.05) between field strengths. Therefore, the *α*_m_-ratio values for these metabolites were estimated separately for each field strength (red and blue lines in Fig 4). For the rest of the metabolites, only one *α*_m_-ratio value per metabolite was estimated using the pooled data (green lines in Fig 4). In both cases, the *α*_m_-ratio was estimated via linear regression of Eq (3).

Table 2 shows the *α*_m_-ratio values estimated in this work, along with literature values as a reference (or the range of values, if available). The putamen mean and standard error of the mean (SEM) metabolite concentrations calculated with the correction method M_2_ are also summarised in Table 2. Additionally, the CRLB values for each field strength are reported.

**Table 2:** Measured metabolites *α*_m_-ratio, literature values and concentration estimated using M_2_ for 3 T and 7 T.

Metabolite	*α*_m_ ± *σ*_α_		*α*_m_ literature values	〈*c*_m,2_〉 ± SEM [mM]		〈CRLB〉 ± SEM [%]	
	3 T	7 T		3 T	7 T	3 T	7 T
Asp	1.15 ± 0.12		0.14 [52]	2.3 ± 0.3	1.7 ± 0.6	15 ± 1.8	27.4 ± 12.3 (*)
GABA	0.26 ± 0.06		0.36–1 [19, 52, 53]	2.2 ± 0.7	2.6 ± 0.8	26.5 ± 9.4	10.8 ± 1.9 (*)
Gln	0.41 ± 0.04	0.44 ± 0.04	0.39–0.57 [52, 54]	5.8 ± 0.7	3.7 ± 0.6	8.7 ± 1.2	6.4 ± 1.7 (*)
Glu	0.61 ± 0.02		0.39–0.84 [28, 52, 54]	10.7 ± 1.2	10.8 ± 0.7	4.2 ± 0.6	2.2 ± 0.4 (*)
GSH	0.94 ± 0.05		0.75 [52]	1.5 ± 0.1	1.5 ± 0.3	10.6 ± 1.2	8.8 ± 2.7 (*)
Ins	1.52 ± 0.14		0.72–1 [54]	5.0 ± 0.7	5.3 ± 0.9	3.2 ± 0.4	3.1 ± 0.7
Lac	2.1 ± 0.3		-	0.5 ± 0.1	0.5 ± 0.2	28.3 ± 11.2	21.7 ± 8.3
NAA	0.88 ± 0.03		0.82–1.05 [28, 52, 54, 55]	10.0 ± 0.6	10.5 ± 0.6	3.0 ± 0.4	2.1 ± 0.3 (*)
NAAG	3.8 ± 1.1		1.86–5.4 [52, 54]	0.9 ± 0.2	0.9 ± 0.2	14.8 ± 3.5	9.3 ± 2.5 (*)
PE	0.54 ± 0.07		-	2.8 ± 0.9	2.7 ± 0.8	20.0 ± 9.7	13.9 ± 7.1
Scyllo	1.1 ± 0.33	-	1 [52]	0.3 ± 0.1	-	22.6 ± 7.1	-
Tau	1.2 ± 0.2		0.5 [52]	1.2 ± 0.2	1.3 ± 0.5	29.6 ± 6.9	19.9 ±11.6 (*)
tCho	1.31 ± 0.09		1.33–1.46 [52, 56, 57]	1.7 ± 0.3	1.6 ± 0.2	4.1 ± 0.5	3.9 ± 0.8
tNAA	1.1 ± 0.04		0.96–1.22 [52, 54]	10.8 ± 0.7	11.5 ± 0.7	2	1.7 ± 0.5 (*)
tCr	0.81 ± 0.04	0.82 ± 0.05	0.77 [52]	8.7 ± 0.8	10.1 ± 0.9	2.5 ± 0.5	2 ± 0.4 (*)
Glx	0.54 ± 0.03	0.57 ± 0.02	0.34–0.8 [28, 52]	16.2 ± 1.7	14.1 ± 0.6	4.0 ± 0.6	2.3 ± 0.5 (*)

The metabolite WM-to-GM concentration ratios, *α*_m_-ratio, estimated in this work are shown in the second and third columns. The standard deviation of *α*_m_, *σ*_α_, was calculated using conventional error propagation based on the fitting errors of *c*_GM_ and *c*_WM_. The fourth column contains the range of literature values obtained by both single-voxel MRS or MRS imaging. Notice that the range of literature *α*_m_-ratio values were calculated in this work using the *c*_WM_ and *c*_GM_ published in the corresponding reference. In these references, the VOIs contained mostly, but not only, WM or GM; therefore, there is always a certain degree of PVE contamination. Mean metabolite concentrations ± SEM [mM] for 3 T (fifth column) and for 7 T (sixth column), were calculated according to method M_2_. CRLB values ± SEM [%] estimated at both 3 T and 7 T are shown in the seventh and eighth columns, respectively. Significant differences in CRLB values at 3 T vs. 7 T are denoted by * (p < 0.05).

**Fig 5:**
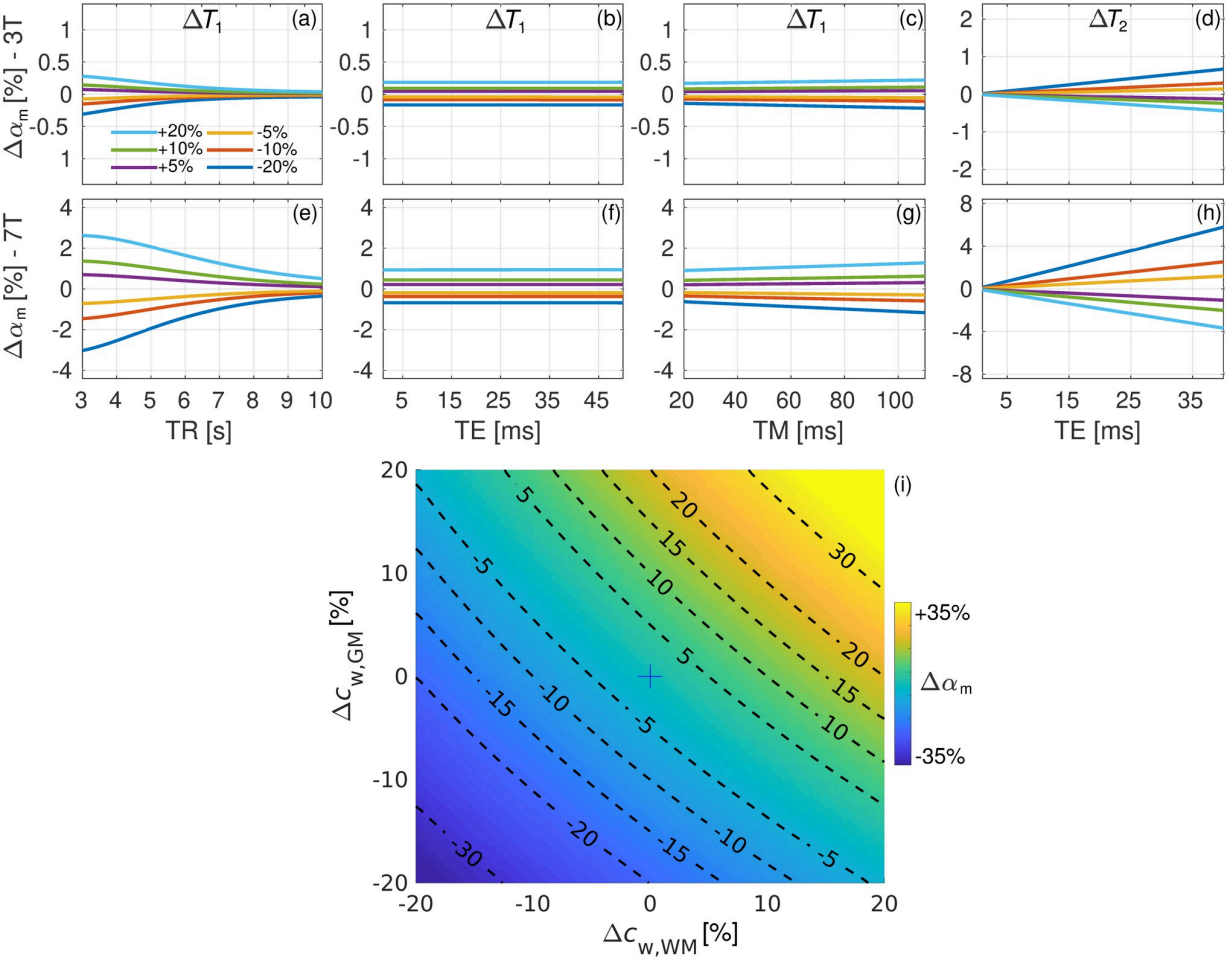
Parameter Δ*α*_m_ as defined in Eq (4) for several values Δ*T*_1_ (a-c, e-g), Δ*T*_2_ (d, h), Δ*c*_w,WM_ and Δ*c*_w,GM_ (i) ranging from -20% to +20%. For the case of relaxation times, a range of TR, TE, and TM values was considered for 3 T (a-d) and 7 T (e-h) scanners.

Fig 5 depicts the parameter Δ*α*_m_ evaluated using Eq (4), where the changes in *b* were evaluated according to Δ*b* = (100% × (*b* − *b*_0_) /*b*_0_. The parameter Δ*α*_m_ was assessed for several values of Δ*T*_1_, Δ*T*_2_, Δ*c*_w,WM_ and Δ*c*_w,GM_ ranging from -20% to +20%. For the case of the water relaxation times, Δ*α*_m_ was evaluated for a range of TE, TR, and TM values (Fig 5a–5h). In the particular case of the water relaxation times and for the TR, TE, and TM used in our experiments, the resulting values of Δ*α*_m_ for the 3 T scanner lie in the range between -0.17% and 0.18%, whereas the for the 7 T scanner the values of Δ*α*_m_ lie in the range between -0.7% and 0.9%. That is to say, based on our experimental parameters, a bias of ±20% in either of the water relaxation times would result in Δ*α*_m_ < 1%. On the contrary, Fig 5i shows that the parameter Δ*α*_m_ is much more sensitive to a change in the tissue water content.

Table 3 shows the Pearson’s correlation coefficients (*ρ*) and p-values of *c*_m,1_ and *c*_m,2_ vs. *φ*_GM_ for each metabolite. As expected, concentrations *c*_m,1_ show a high correlation (p < 0.05) with *φ*_GM_, for metabolites whose WM and GM concentrations are notoriously different. However, *c*_m,2_ concentrations show no correlation with *φ*_GM_ (p > 0.05) in any metabolites, highlighting the ability of M_2_ to remove the dependence of the concentration on *φ*_GM_.

**Table 3:** Summary of Pearson’s correlation coefficients analysis for M_1_ and M_2_.

Metabolite	*ρ* for *c*_m,1_ vs. *φ*_GM_		*ρ* for *c*_m,2_ vs. *φ*_GM_	
	3 T	7 T	3 T	7 T
Asp	-0.16 (p = 0.26)		-0.006 (p = 0.97)	
GABA	0.68 (p < 0.001)		0.03 (p = 0.86)	
Gln	0.87 (p < 0.001)	0.88 (p < 0.001)	0.02 (p = 0.94)	0.01 (p = 0.96)
Glu	0.81 (p < 0.001)		0.03 (p = 0.84)	
GSH	0.13 (p = 0.36)		0.002 (p = 0.99)	
Ins	-0.51 (p < 0.001)		0.009 (p = 0.95)	
Lac	-0.62 (p < 0.001)		0.005 (p = 0.97)	
NAA	0.46 (p < 0.001)		0.006 (p = 0.97)	
NAAG	-0.7 (p < 0.001)		0.06 (p = 0.68)	
PE	0.47 (p < 0.001)		0.03 (p = 0.81)	
Scyllo	-0.07 (p = 0.79)	-	-0.004 (p = 0.99)	-
Tau	-0.15 (p = 0.32)		-0.0008 (p = 1.0)	
tCho	-0.44 (p < 0.001)		-0.004 (p = 0.98)	
tNAA	-0.31 (p = 0.025)		0.001 (p = 0.99)	
tCr	0.531 (p = 0.0036)	0.52 (p = 0.0077)	0.009 (p = 0.96)	0.009 (p = 0.96)
Glx	0.84 (p < 0.001)	0.92 (p < 0.001)	0.03 (p = 0.89)	0.04 (p = 0.86)

Pearson’s correlation coefficients (*ρ*) of *c*_m,1_ and *c*_m,2_ vs. *φ*_GM_ and the corresponding p-values, for 3 T and 7 T data. Correlation analysis was performed for pooled (3 T and 7 T) data except for Gln, tCr and Glx, given their significant difference in *c*_m,1_ distributions between the scanners based on the ANCOVA analysis.

### 3.4 Effect of a bias in α_m_ values in method M_2_

**Fig 6:**
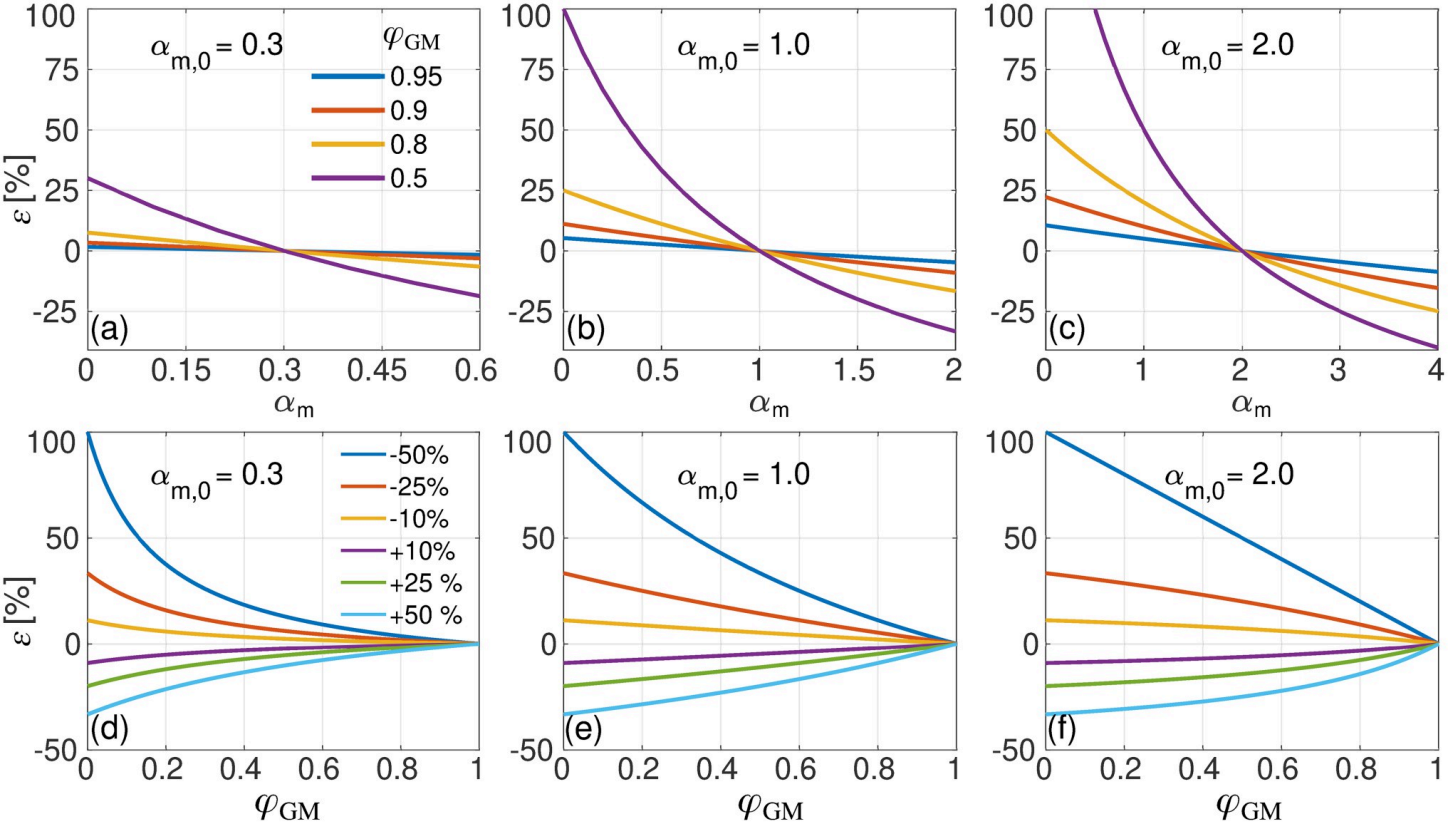
(a-c) Evaluation of the error, *ε* (Eq 5), in the metabolite concentration as estimated using M_2_ vs. *α*_m_, assuming that the used fraction *α*_m_ deviates from the actual fraction *α*_m,0_ = 0.3 (a), 1.0 (b), and 2.0 (c). Several values of the relative fraction *φ*_GM_ are shown. (d-f) Evaluation of the error, *ε*, vs. *φ*_GM_, for several values of *α*_m_, assuming true values of *α*_m,0_ = 0.3 (d), 1.0 (e) and 2.0 (f).

Fig 6a–6c shows the simulated error, *ε* (Eq 5), as a function of the concentration ratio, *α*_m_, used in Eq (2). Here, actual concentration ratios *α*_m,0_ = 0.3 (a), 1.0 (b) and 2.0 (c) are assumed. The error is shown for relative GM fractions with *φ*_GM_ ranging from 0.5 to 0.95. Fig 6d–6f depicts *ε* as a function of *φ*_GM_ assuming actual concentration ratios of *α*_m,0_ = 0.3 (d), 1.0 (e) and 2.0 (f). Several values of *α*_m_ are considered, ranging from an underestimation equal to -50% to an overestimation of +50% of the actual concentration ratio *α*_m,0_. We observed that *ε* increases as the relative fraction *φ*_GM_ decreases. It is also clear that, for a given *α*_m,0_, the error tends to be smaller if *α*_m_ > *α*_m,0_ than if *α*_m_ < *α*_m,0_ by the same amount. In other words, it is preferable to overestimate the concentration ratio used in M_2_ than to underestimate it (which was also previously observed by Harris et al. [19]). Note also that the error *ε* tends to increase more rapidly with decreasing *φ*_GM_ for the case *α*_m,0_ > 1 (Fig 6f) compared to the case *α*_m,0_ < 1 (Fig 6d). That is to say, metabolites with *α*_m,0_ > 1 tend to be more volatile than those with *α*_m,0_ < 1 when quantified using method M_2_ (Eq (2)).

### 3.5 Comparison of correction methods and field strengths

**Fig 7:**
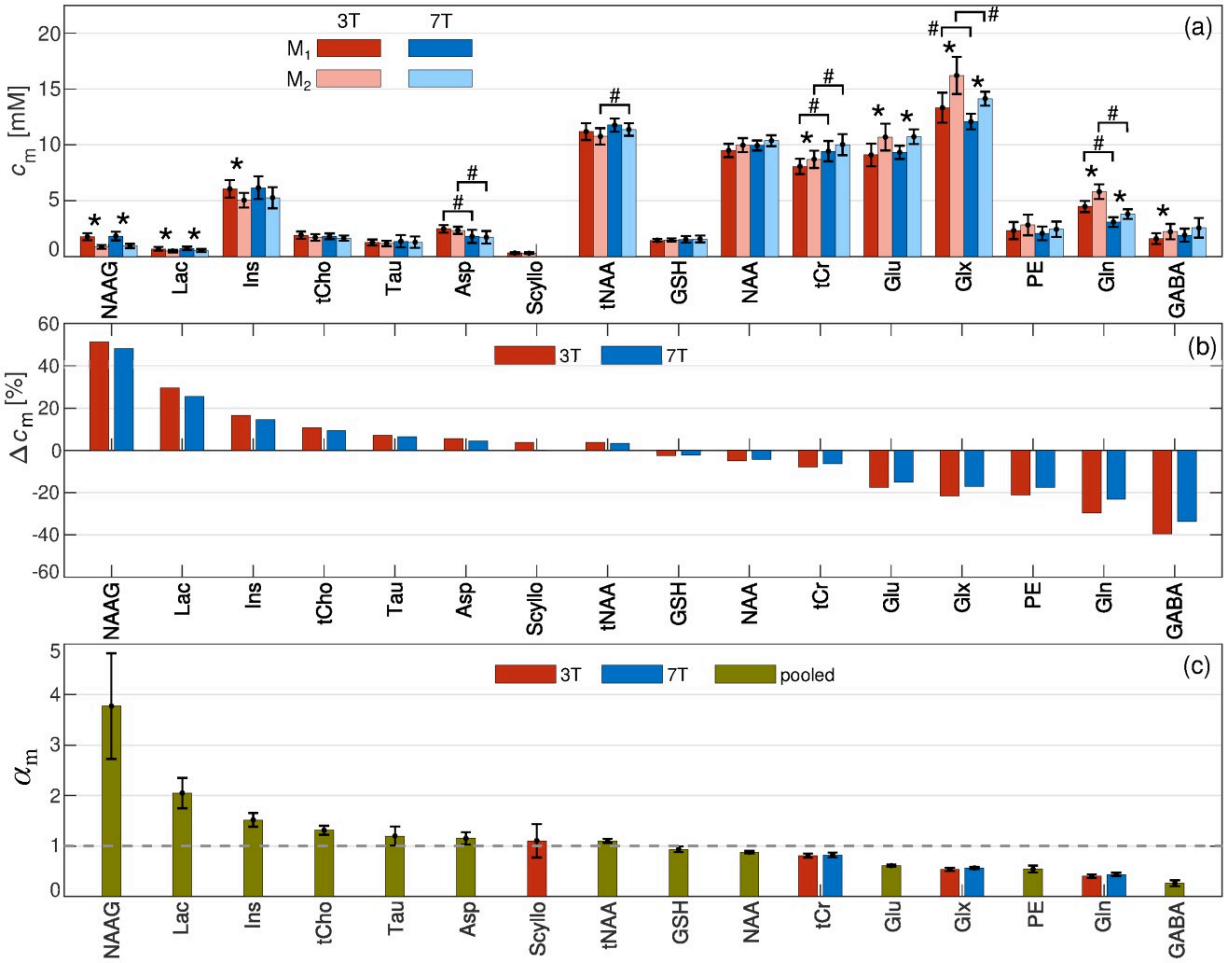
(a) Comparison of the correction methods M_1_ and M_2_ for 3 T (dark and light red) and 7 T (dark and light blue) scanners. The mean and SEM of the metabolite concentrations across the subjects of the putamen group are shown. A two-sample *t*-test analysis was performed to compare M_1_ with M_2_ (*, p < 0.05). (b) Relative difference in metabolite concentrations between M_1_ and M_2_ methods, Δ*c*_m_. (c) *α*_m_-ratio values were calculated using either pooled data (green), only 3 T data (red) or only 7 T data (blue). Metabolites are ordered in such a way that *α*_m_ appears in descending order from left to right. For the case of Gln, tCr and Glx the *α*_m_-ratio values from 7 T are used for ordering purposes.

Fig 7a shows a comparison of the mean and SEM of the metabolite concentrations taken over the whole putamen group, calculated using methods M_1_ and M_2_, for 3 T (red) and 7 T (blue) scanners. In the case of metabolites where *α*_m_ differed significantly from unity, we observed that M_2_ led to significantly different concentrations (*, p < 0.05) compared to M_1_. It is, however, important to note that another factor influencing the significance of the difference between M_1_ and M_2_ is the inter-subject variability of the metabolite concentration. Notice that the M_2_ method for the case of Gln, tCr, and Glx was evaluated using the corresponding separate *α*_m_-ratio values (either 3 T or 7 T), based on the observed significant difference of the *c*_m,1_ concentration distributions between field strengths. For the rest of the metabolites, the *α*_m_-ratio values obtained using pooled data were used. Regarding the comparison between the scanners, Asp, tNAA, tCr, Glx, and Gln showed a significant difference between the field strengths (#, p < 0.05) for both correction methods. The relative difference between the concentrations calculated with M_2_ and M_1_, Δ*c*_m_ = 100% × (*c*_m,2_ − *c*_m,1_)/*c*_m,1_, is plotted in Fig 7b. Fig 7c shows the plot of the corresponding *α*_m_-ratio values for the whole metabolic profile.

## 4. Discussion

We have provided a thorough quantification of the metabolic profile of the human putamen in a cohort of healthy, elderly subjects. Although the features covered by the M_1_ method have already been broadly discussed in the literature [13, 25, 28, 33, 58], the *α*_m_-ratio method has only been investigated for the case of GABA in a few works [19, 22, 59]. Hence, our secondary goal focused on investigating the *α*_m_-ratio method, with the putamen being the anatomical target. Finally, concentrations quantified at field strengths of 3 T and 7 T were compared as a means of validation.

The data quality for the 3 T and 7 T scanners, as assessed via the linewidth of the unsuppressed water peaks, reflected a strong, positive correlation between the scanners (Fig 2). Furthermore, good spectral quality was achieved throughout the whole study, which is evident in the average (Fig 3) and individual spectra (S2 Fig). Additionally, better B0 shimming was achieved in the case of the occipital, parietal and frontal WM VOIs compared to the putamen VOI, as observed in Fig 2. The most likely explanation is that the difference in the magnetic susceptibility between the putamen and the surrounding WM matter creates large B0 field distortions [60]. In this work, we have utilised FASTESTMAP for B0 shimming, which optimises shim coils up to second-order. Although it has been demonstrated that the use of higher-order terms in the shimming of the frontal and occipital cortex plays only a marginal role [61], the effect of higher shim orders in other regions, e.g. the putamen, may be more substantial, and therefore, further investigation is required.

The use of STEAM enabled the data acquisition at ultra-short TE, which in turn has the advantage of providing i) high SNR spectra and ii) low spectral distortion of multiplets. The combination of these features facilitated the reliable detection and quantification of 12 metabolites and four total concentrations, as reflected by the low CRLB values [23, 24, 39]. Despite the fact that the mean SNR values were comparable at both fields (as a result of different VOI sizes and number of averages), our results also show that the CRLB values are, in general, lower at 7 T than at 3 T for the putamen [9, 24, 62]. This demonstrates that the simplification of spectral patterns and lower peak overlap at higher field strengths indeed play an important role in the quantification precision, even for comparable SNR values [24]. More specifically, the reduction in the CRLB values for the “mostly singlet” metabolites (e.g. NAA, tCr and tCho) [63] was in the range -5% to -30% for 7 T compared to 3 T. Conversely, the decrease in the CRLB values for the J-coupled metabolites (e.g. GABA, Glu, Gln, Lac, NAAG, PE and Tau) was broader, ranging from -17% for GSH and going up to -48% for Glu and -60% for GABA. In general, the milder improvement in quantification precision with increasing field strength for “mostly singlet” metabolites compared to the large improvement seen in the case of J-coupled metabolites has been previously demonstrated using Monte Carlo simulations [63] and *in vivo* experiments [24, 62, 64]. Furthermore, the behaviour for the particular cases of Ins, which showed almost no decrease in the CRLB values with increasing field strength (-3%), and Asp, which showed increasing CRLB values, has also been observed in simulations [63] and *in vivo* experiments [24, 62, 64]. Finally, Scyllo was only reliably detected at 3 T, which was similar to other works in the literature where the CRLB worsened with increasing field strength [24, 62, 65] and is most likely due to its intrinsically low concentration.

Conversely, a drawback of short-TE sequences is the presence of strong macromolecular background peaks that complicate spectral fitting [66]. To overcome this, we have included in the LCModel basis sets the macromolecular spectra acquired using the inversion recovery STEAM sequence measured using similar experimental parameters [24, 46]. The validity of using a general macromolecular spectrum is based on the fact that the difference in such spectra between tissue types is negligible [66]. Nevertheless, an investigation using age- and region-specific macromolecular spectra in the basis set is worth considering for future studies.

The short TE and relatively long TR used in our work allowed us to ameliorate a possible bias in *α*_m_ due to any potential inaccuracy in the relaxation times used for water and metabolites in Eqs (1) and (2). Indeed, it has been shown that ageing induces changes in the water relaxation times [67–71] as well as in the metabolite relaxation times [72–74]. Hence, the use of single literature values may pose a limitation when studying groups comprising a broad range of ages. The age range covered in this work was 53 to 80. An examination of the literature revealed that, in this age range, the water *T*_1_ in WM can increase by up to roughly 10% in WM [67], whereas the increment in *T*_2_ can be up to nearly 8% [71]. Regarding the putamen, the literature suggests a milder decrease with age of nearly 2% in *T*_2_ [69, 71]. On the contrary, there is some conflicting evidence regarding the age-related changes in the putamen *T*_1_, with some works demonstrating increments with age of roughly 18% [67] while others report no significant changes [68] or even a decrease with age [69]. Given our experimental timings, the analysis shown in Fig 5a–5h demonstrates that a bias of ±20% in the water relaxation times (which englobes the former reported changes and other possible sources of bias) would result in Δ*α*_m_ < 1% from the values reported here. However, it is evident that in the case of experiments using short TR and/or long TE, the use of age- and anatomy-matched relaxation time values is crucial. In contrast, Fig 5i shows a stronger dependence of Δ*α*_m_ versus a potential bias in *c*_w,WM_ or *c*_w,GM_. However, Neeb et al. [29] reported no age dependency for *c*_w,WM_, whereas for *c*_w,GM_ a decrease of 0.034% per year was observed. That gives a total reduction of roughly -1.2% in the age range of 53 to 80. Moreover, Taubert et al. [75] also observed a slight reduction in water content in the putamen, although the numbers were not published. Hence, although the effect of ageing on *α*_m_ via *c*_w,WM_ or *c*_w,GM_ in the studied age range is not expected to be substantial, its investigation in a broader age range should be carefully considered.

The impact of the intrinsic assumption made in Eqs (1) and (2) that the metabolite relaxation times in WM and GM are equal has been previously addressed by Gasparovic et al. [76]. In their work, the authors demonstrate that as the correct equation for accounting for differences in the metabolites relaxation times between WM and GM contains two unknowns, it is not explicitly solvable under conventional experimental designs. Nevertheless, Gasparovic and colleagues use the equation to examine the impact of differing metabolite relaxation times between WM and GM on the estimated concentration for different experimental parameters. For the particular case of NAA at 3 T, they show that an assumption of equal T1/T2 metabolite relaxation times can lead to an error of the order of 6.5–7.8% in the estimated concentration using TR = 1.5 s. and TE = 0.144 s. However, the error was drastically reduced to 0.5% for TR = 6 s and TE = 0.006.

In order to estimate the *α*_m_-ratio values, we first utilised ANCOVA to compare the *c*_m,1_ vs. *φ*_GM_ distributions at 3 T and 7 T. A lack of significance in the difference indicated that the *c*_m,1_ vs. *φ*_GM_ distributions could be pooled for the estimation of a single *α*_m_-ratio. On the contrary, a significant difference in the *c*_m,1_ vs. *φ*_GM_ distributions suggests that the *α*_m_-ratios at 3 T and 7 T could diverge due to reasons other than just differences in the tissue voxel composition. However, even though the *c*_m,1_ vs. *φ*_GM_ distributions were significantly different for Gln, tCr, and Glx, the *α*_m_-ratio values were comparable. The *α*_m_-ratios measured here were generally in line with the *α*_m_-ratio values calculated using literature WM and GM metabolite concentrations (Table 2), although some discrepancies were observed. In the case of GABA, for instance, we report a ratio *α*_GABA_ = 0.26 ± 0.06 whereas the literature range is 0.35–1.0 [19, 52, 53]. This observation is in line with a study by Fahn et al. [77], where it was also reported that the highest GABA levels were found in subcortical regions (e.g. basal ganglia) compared to the cortex in rhesus monkeys. Another example is Ins, for which we obtained a ratio of *α*_Ins_ = 1.52 ± 0.14, whereas the literature range is 0.44–1.0 [54]. One explanation for these discrepancies could be that the literature values were attained using VOIs positioned in WM and cortical GM, whereas in our study, the VOI mainly contained subcortical GM [52, 54]. Another possible factor is attributed to the age-dependency of the concentration of some metabolites [78–81]. Finally, another aspect could be ascribed to the tissue segmentation approach [19]. However, an investigation of different segmentation approaches was beyond the scope of this work.

Accounting for the *α*_m_-ratio has been shown to have important implications in studies where the subject group includes significant differences in the tissue voxel composition due to brain atrophy, for example [21, 22, 59]. Nevertheless, previous studies have focused on the quantification of GABA, and, to the best of our knowledge, the method has not been applied to other metabolites. In the present work, we have provided a quantification of the *α*_m_-ratio for the whole metabolic profile of the human putamen and surrounding WM. For some metabolites, such as GABA, Gln, Glx, Glu and PE, concentrations in the putamen are clearly larger than in the surrounding WM. Therefore, the inclusion of the *α*_m_-ratio in the quantification via M_2_ has a significant effect on the evaluated concentration (Fig 7) [19]. Metabolites, such as tCr, NAA, GSH, Asp, tNAA, Scyllo, Tau, and tCho showed *α*_m_-ratios close to unity, and consequently, no significant differences between *c*_m,2_ and *c*_m,1_ were observed except in tCr, which differed significantly between the methods in 3 T data only. For the case of Ins and Lac, the concentration in WM was clearly higher than in GM. However, the case of Ins only showed a significant difference between *c*_m,2_ and *c*_m,1_ in 3 T data. This can be explained by the fact that the WM fraction was higher in the 3 T VOI than in the 7 T VOI. Finally, NAAG also showed a higher concentration in WM compared to GM, which resulted in a significant difference between *c*_m,2_ and *c*_m,1_ at both field strengths. More generally, the ability of M_2_ to remove the dependency of the estimated putamen metabolite profile on the partial volume was further demonstrated via the Pearson’s correlation analysis (Table 3). While metabolites showing *α*_m_-ratio values significantly different from unity showed a strong, significant correlation with *φ*_GM_ when quantified with M_1_, the correlation was completely absent when M_2_ was used. Hence, identifying metabolites for which *α*_m_-ratio deviates significantly from unity is important.

Contrary to several works in the literature devoted to investigating the advantages and pitfalls of MRS techniques with increasing B0 strengths, which normally utilise matched voxel sizes and/or similar protocol parameters [9, 24, 62, 65], our study included different protocols, especially regarding the voxel size (and therefore the tissue voxel composition). This feature served to investigate the quantification approach when comparing method M_2_ to M_1_. This validation approach was inspired by the work of Dhamala et al. [25], in which the concentrations measured using three different MRS sequences with different protocol parameters were compared to validate the metabolite concentrations. The rationale behind this approach is that comparable results obtained using different approaches give more confidence in the absolute concentration achieved. Similarly, indistinguishable concentrations measured using different field strengths and/or protocol parameters (including VOI tissue composition) should strengthen the soundness of the quantified putamen metabolic profile. In our work, the observed difference in Asp, tNAA, tCr, Glx and Gln concentrations between 3 T and 7 T when using method M_1_, was still observed for method M_2_. Therefore, one can conclude that these differences are not due to the different tissue compositions of the voxels at 3 T and 7 T and other factors, such as different chemical shift displacement errors between the scanners, should be considered.

A context in which the *α*_m_-ratio deserves special attention is functional MRS (fMRS). Given that the observed changes in metabolite concentrations due to activation primarily take place in GM, it is expected that, as per the definition of *α*_m_, changes in *α*_m_ will also occur as a result of brain activity. Starting from the derivation of Eq (2) [19], it is trivial to show that the difference between the measured metabolite concentration during activation and the resting-state condition is Δ*c* = *f*_GM_ Δ*c*_GM_ and therefore |Δ*c*| ≤ |Δ*c*_GM_|. Hence, the change in the metabolite concentration between activation and resting state, as assessed using Eq (2), will show a larger or equal magnitude compared to the change in the measured (i.e. VOI-specific) metabolite concentration. In other words, Eq (2) has the effect of magnifying the change in metabolite concentrations due to brain activation compared to the VOI-specific measured change (Eq (1)). In the particular case of our study, the MRS experiments were performed under resting state conditions, and therefore, there are no physiological reasons to expect variations in the *α*_m_-ratio. However, studies have shown that in the presence of some stimuli, the concentration of metabolites, e.g. Lac, can fluctuate as much as 30% within the VOI [82]. Therefore, a study fully devoted to the investigation of the *α*_m_-ratio within the framework of fMRS is worth considering for future studies.

It is worth mentioning that we have not made use of the tissue-correction approach normalised to the study-specific voxel composition, as originally proposed by Harris et al. (Eq (6) in reference [19]). The reason for this is that we were interested in specifically assessing the absolute metabolic profile of the putamen. That is to say, the metabolic profile of the putamen must not depend on the MRS tissue voxel composition. However, tissue composition normalisation is well advised for cases such as group comparison, especially when brain atrophy is involved [19]. Therefore, given the importance of the former method, a thorough understanding of the *α*_m_-ratio for the whole metabolic profile for the different brain regions becomes paramount and emphasises the importance of our results.

## 5. Limitations

A limitation of this work is the fact that the metabolite basis sets used in LCModel were simulated using ideal RF pulses. However, while real RF pulses with localisation gradients are preferable, the improvement is not expected to be substantial for the case of STEAM, as shown by Kaiser et al. [83].

A further important limitation of this work is that the tissue water concentration was assumed based on literature values, which normally correspond to the case of healthy, young volunteers. However, it has been shown that the tissue water concentration can be altered, not only in the case of brain pathologies, e.g. hepatic encephalopathy [84], stroke and tumour [85], and cirrhosis [86], but also due to age [29]. Furthermore, the tissue water concentration depends on brain anatomy [87]. Although the impact of age on tissue water content is rather mild [29], increases of nearly 7% in stroke and roughly 10%-15% in tumour tissue have been reported [85]. These changes would result in large deviations in the estimated metabolite concentration and *α*_m_-ratios, as shown in Fig 5i. This drawback could be overcome in future studies by introducing a water mapping technique in the study protocol to assess region- and subject-specific water concentrations [15, 27, 29, 87–91]. More generally, the ideal experimental setup should pursue the determination of subject- and tissue-specific water relaxation times and concentration [15] as well as metabolite relaxation times.

## 6. Conclusions

We have investigated a method for the quantification of the neurochemical profile of the brain using MRS, which accounts for differences in the metabolite concentrations of the different tissues comprised in the voxel. The method was applied to quantify the neurochemical profile of the human putamen in a cohort of healthy elderly subjects using STEAM MRS at 3 T and 7 T. The data were then compared to the metabolite concentrations obtained using the conventional correction approach that assumes equal concentration in all tissues. We demonstrate that accounting for differences in the metabolite concentrations between WM and GM leads to significant differences in the estimated metabolite concentration provided that *α*_m_ significantly differs from unity. Furthermore, the investigated method was able to remove any dependence of the putamen metabolite concentration on the tissue voxel composition. Finally, not only have we provided a quantification of the neurochemical profile of the human putamen, but also the *α*_m_-ratio of the metabolite concentration profile with the surrounding WM. Our data may potentially serve as a reference for the classification of the degree of metabolic changes in the putamen within the framework of neurodegenerative diseases.

## Supporting information

S1 Fig VOI positioning in the occipital, parietal and frontal WM at 3 T and 7 T. (DOCX)

S2 Fig MRS spectra for each of the subjects from the putamen group. Dotted lines denote the subjects for whom the spectra were skipped due to a linewidth greater than 0.07 ppm.
(DOCX)

S1 Table Mean metabolite concentrations as outputted by LCModel without further corrections. (DOCX)

S2 Table Metabolite relaxation times for 3 T and 7 T. (DOCX)

## Abbreviations

1H-MRS: proton magnetic resonance spectroscopy
Ala: alanine
Asc: ascorbate
Asp: aspartate
Cr: creatine
CRLB: Cramér-Rao lower bound
CSF: cerebrospinal fluid
FWHM: full width at half maximum
GABA: γ-aminobutyric acid
Glc: glucose
Gln: glutamine
Glu: glutamate
Glx: glutamate + glutamine
GM: grey matter
GPC: glycerophosphorylcholine
GSH: glutathione
Ins: *myo*-inositol
Lac: lactate
MM: macromolecules
NAA: *N*-acetylaspartate
NAAG: *N*-acetylaspartylglutamate
PCh: phosphorylcholine
PCr: phosphocreatine
PE: phosphorylethanolamine
PVE: partial volume effect
RF: radiofrequency
Scyllo: *scyllo*-inositol
SEM: standard error of the mean
SNR: signal-to-noise ratio
STEAM: stimulated echo acquisition mode
Tau: taurine
tCho: total choline
tCr: total creatine
TE: echo time
TM: mixing time
tNAA: total NAA
TR: repetition time
VOI: volume of interest
WM: white matter
