# Supplementary material for: Quantification of the neurochemical profile of the human putamen using STEAM MRS in a cohort of elderly subjects at 3 T and 7 T: Ruminations on the correction strategy for the tissue voxel composition
Source: PLoS One. 2023 Jun 2;18(6):e0286633. doi: 10.1371/journal.pone.0286633 (PMC10237501; doi:10.1371/journal.pone.0286633)
Supplement: S1 Fig — (DOCX) [file pone.0286633.s001.docx]

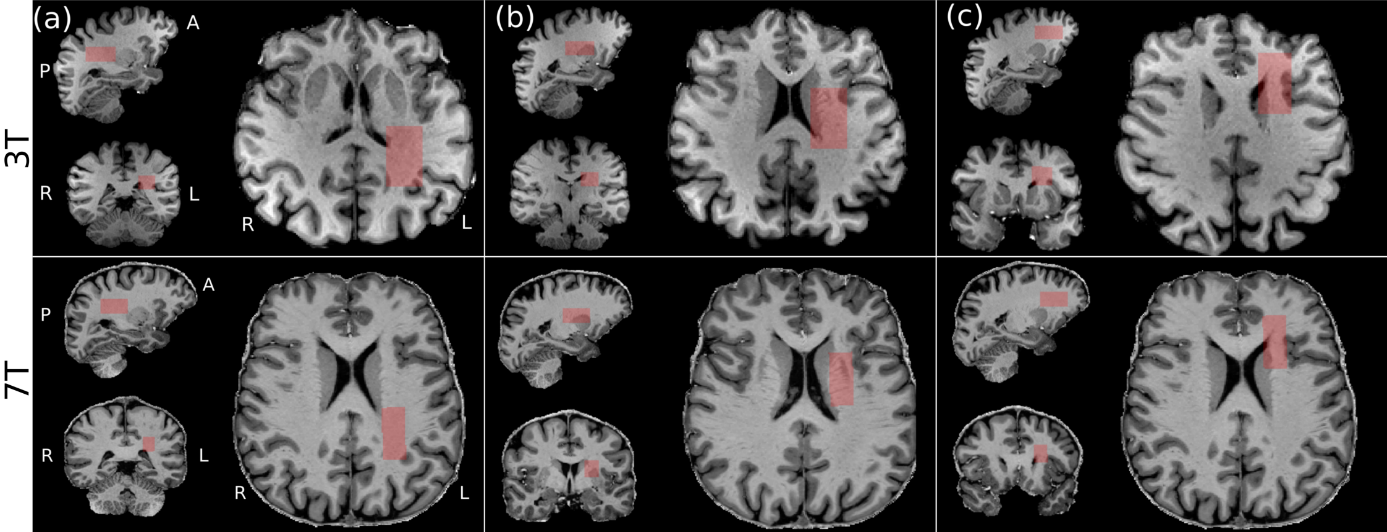


**S1 Fig.** VOI positioning of one of the five extra subjects used for the assessment of the $\alpha_{m}$ concentration ratio (Eq. 3). VOIs were located in the occipital WM (a), parietal WM (b), frontal WM (c) for 3 T (top panel) and 7 T (bottom panel).
