# Supplementary figures and images for: Quantification of the neurochemical profile of the human putamen using STEAM MRS in a cohort of elderly subjects at 3 T and 7 T: Ruminations on the correction strategy for the tissue voxel composition
Source: PLoS One. 2023 Jun 2;18(6):e0286633. doi: 10.1371/journal.pone.0286633 (PMC10237501; doi:10.1371/journal.pone.0286633)

**
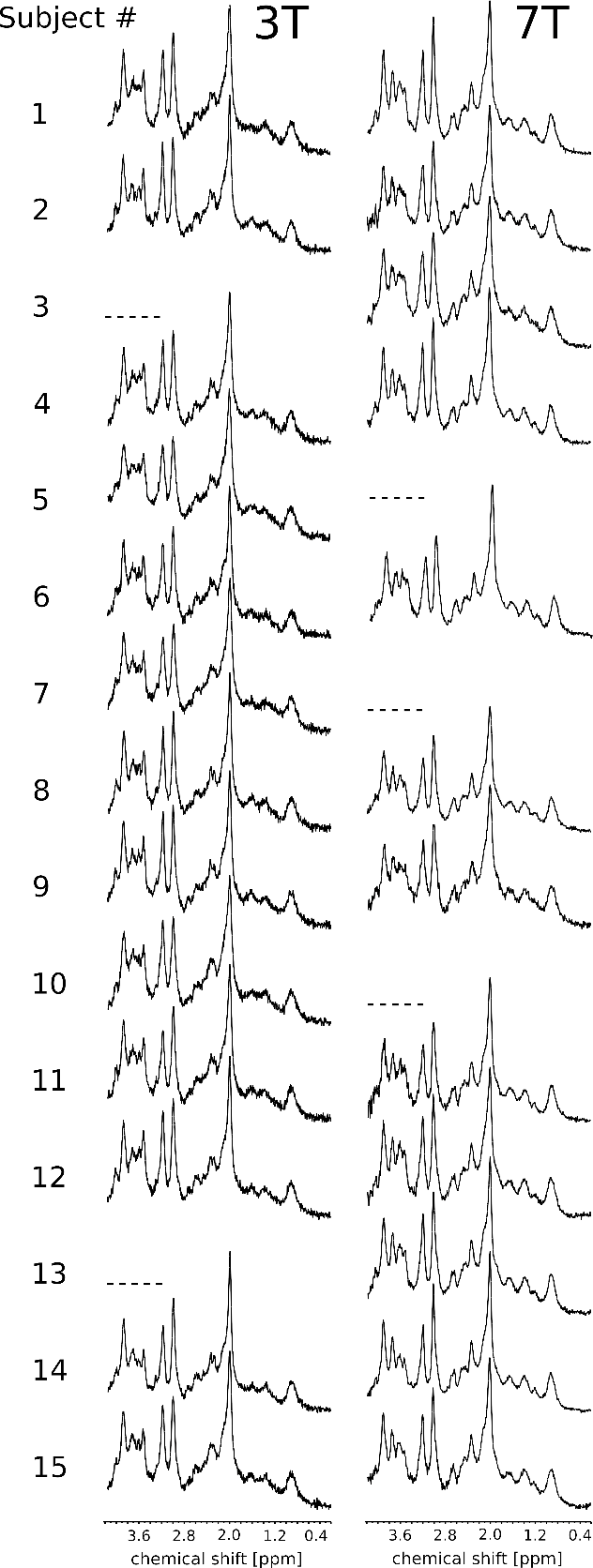
**

**S2 Fig.** MRS spectra for each subject in the putamen group, for the 3 T and 7 T scanners.

Supplement: S2 Fig — Dotted lines denote the subjects for whom the spectra were skipped due to a linewidth greater than 0.07 ppm. (DOCX) [file pone.0286633.s002.docx]
