# Supplementary material for: Quantification of the neurochemical profile of the human putamen using STEAM MRS in a cohort of elderly subjects at 3 T and 7 T: Ruminations on the correction strategy for the tissue voxel composition
Source: PLoS One. 2023 Jun 2;18(6):e0286633. doi: 10.1371/journal.pone.0286633 (PMC10237501; doi:10.1371/journal.pone.0286633)
Supplement: S1 Table — (DOCX) [file pone.0286633.s003.docx]

**S1 Table. Mean metabolite concentrations as outputted by LCModel without further corrections.**

| Metabolite | conc ± *σ*_conc_ | |
| --- | --- | --- |
|  | 3T | 7T |
| Asp | 1.51 ± 0.2 | 1.15 ± 0.37 |
| GABA | 0.96 ± 0.3 | 1.23 ± 0.35 |
| Gln | 2.8 ± 0.33 | 1.92 ± 0.35 |
| Glu | 5.63 ± 0.64 | 6 ± 0.35 |
| GSH | 0.83 ± 0.08 | 0.95 ± 0.2 |
| Ins | 3.85 ± 0.52 | 4.01 ± 0.61 |
| Lac | 0.41 ± 0.11 | 0.43 ± 0.12 |
| NAA | 5.92 ± 0.39 | 6.53 ± 0.36 |
| NAAG | 1.07 ± 0.2 | 1.21 ± 0.3 |
| PE | 1.41 ± 0.46 | 1.44 ± 0.45 |
| Scyllo | 0.19 ± 0.06 | - |
| Tau | 0.77 ± 0.16 | 0.85 ± 0.36 |
| tCho | 1.2 ± 0.21 | 1.16 ± 0.15 |
| tNAA | 6.99 ± 0.51 | 7.73 ± 0.55 |
| tCr | 4.97 ± 0.44 | 6.07 ± 0.54 |
| Glx | 8.42 ± 0.88 | 7.92 ± 0.41 |
