# Supplementary material for: Quantification of the neurochemical profile of the human putamen using STEAM MRS in a cohort of elderly subjects at 3 T and 7 T: Ruminations on the correction strategy for the tissue voxel composition
Source: PLoS One. 2023 Jun 2;18(6):e0286633. doi: 10.1371/journal.pone.0286633 (PMC10237501; doi:10.1371/journal.pone.0286633)
Supplement: S2 Table — (DOCX) [file pone.0286633.s004.docx]

**S2 Table. Metabolite relaxation times for 3 T and 7 T.**

| *T*_1_ [s] | 7 T | region | Occipital [4] (Ins) | PCC [5] | PCC [5] (Cr) | Occipital [4] | PCC [5] | Occipital [4] | Occipital [4] | Parietal-occipital GM [6] | Occipital [4] | Occipital [4] | Occipital [4] (Ins) | Occipital [4] | Occipital [4] | Occipital [4] (NAA) | Occipital [4] (tCho) | Occipital [4] | Occipital [4] | not reliably detected | Occipital [4] | Putamen [7]  Putamen [7]   - [7] |
| --- | --- | --- | --- | --- | --- | --- | --- | --- | --- | --- | --- | --- | --- | --- | --- | --- | --- | --- | --- | --- | --- | --- |
|  |  |  | 1.28 | 1.75 | 1.75 | 1.74 | 1.33 | 1.64 | 1.61 | 1.68 | 1.14 | 1.28 | 1.28 | 1.83 | 1.21 | 1.83 | 1.51 | 1.51 | 1.31 |  | 2.15 | 1.7 (GM)  1.2 (WM)  4.425 (CSF) |
|  | 3 T | region | Thalamus [1] (Ins) | Thalamus [1] | Thalamus [1] (Cr) | Several [2] | PCC [8] | Occipital [9] (Glx) | Occipital [9] | Occipital [9] | Motor [10] | Thalamus [1] | Thalamus [1] (Ins) | Thalamus [1] | Thalamus [1] (NAA) | Occipital [9] | Thalamus [1] (tCho) | Thalamus [1] | Thalamus [1] (Ins) | Thalamus [1] (Ins) | Thalamus [1] (Ins) | Thalamus [1]  Occipital [1]  - [7] (at 4T) |
|  |  |  | 1.11 | 1.45 | 1.45 | 1.47 | 1.31 | 1.2 | 1.27 | 1.2 | 0.45 | 1.11 | 1.11 | 1.57 | 1.57 | 1.47 | 1.38 | 1.38 | 1.11 | 1.11 | 1.11 | 1.11 (GM)  1.2 (WM)  4.472 (CSF) |
| *T*_2_ [ms] | 7 T | region | Basal ganglia [3] | PCC [5] | PCC [5] (Cr) | Basal ganglia [3] | PCC [11] | Frontal GM/right-frontal WM [12] | Basal ganglia [3] | Parietal-occipital GM [6] | Several [3] | Basal ganglia [3] | Basal ganglia [3] | Basal ganglia [3] | Basal ganglia [3] (NAA) | Basal ganglia [3] (NAA) | Basal ganglia [3] (tCho) | Basal ganglia [3] | Basal ganglia [3] (Ins) | not reliably detected | Basal ganglia [3] | Basal ganglia [3]  WM [7]  - [13] |
|  |  |  | 87 | 109 | 109 | 90 | 63 | 130 | 88 | 274 | 79 | 87 | 87 | 130 | 130 | 130 | 121 | 121 | 87 |  | 85 | 41.2 (GM)  55 (WM)  141 (CSF) |
|  | 3 T | region | Occipital [14] | Occipital [14] | Occipital [14] (Cr) | Basal ganglia [2] | Occipital [14] | ACC [15] | Prefrontal [16] | GM [17] | Occipital [14] | Occipital [14] | Occipital [14] | Occipital [14] | Occipital [14] | Basal ganglia [2] | Occipital [14] | Occipital [18] | Occipital [14] | Occipital [14] | Occipital [14] | Prefrontal [16]  Prefrontal [16]  - [17] |
|  |  |  | 90 | 144 | 144 | 143 | 75 | 145 | 122 | 200 | 77 | 229 | 99 | 263 | 107 | 221 | 213 | 207 | 86 | 107 | 102 | 79 (GM)  79 (WM)  502 (CSF) |
|  |  | Metabolite | Asp | Cr | PCr | tCr | GABA | Gln | Glu | Glx | GSH | Ins | Lac | NAA | NAAG | tNAA | GPC | tCho | PE | Scyllo | Tau | H2O |

A summary of the literature values for longitudinal (*T*_1_) and transverse (*T*_2_) relaxation times at 3 T and 7 T used for quantification. The corresponding brain regions are additionally included.

**References**

1. Ethofer T, Mader I, Seeger U, Helms G, Erb M, Grodd W, et al. Comparison of Longitudinal Metabolite Relaxation Times in Different Regions of the Human Brain at 1.5 and 3 Tesla. Magn Reson Med. 2003;50(6):1296–301.

2. Träber F, Block W, Lamerichs R, Gieseke J, Schild HH. 1H Metabolite Relaxation Times at 3.0 Tesla: Measurements of T1 and T2 Values in Normal Brain and Determination of Regional Differences in Transverse Relaxation. J Magn Reson Imaging. 2004;19(5):537–45.

3. Marjańska M, Auerbach EJ, Valabrègue R, Van de Moortele PF, Adriany G, Garwood M. Localized 1H NMR spectroscopy in different regions of human brain in vivo at 7T: T 2 relaxation times and concentrations of cerebral metabolites. NMR Biomed. 2012;25(2):332–9.

4. Xin L, Schaller B, Mlynarik V, Lu H, Gruetter R. Proton T1 relaxation times of metabolites in human occipital white and gray matter at 7 T. Magn Reson Med. 2013;69(4):931–6.

5. Andreychenko A, Klomp DWJ, De Graaf RA, Luijten PR, Boer VO. In vivo GABA T2 determination with J-refocused echo time extension at 7 T. NMR Biomed. 2013;26(11):1596–601.

6. Penner J, Bartha R. Semi-LASER 1H MR spectroscopy at 7 Tesla in human brain: Metabolite quantification incorporating subject-specific macromolecule removal. Magn Reson Med. 2015;74(1):4–12.

7. Rooney WD, Johnson G, Li X, Cohen ER, Kim SG, Ugurbil K, et al. Magnetic field and tissue dependencies of human brain longitudinal 1H2O relaxation in vivo. Magn Reson Med. 2007;57(2):308–18.

8. Puts NAJ, Barker PB, Edden RAE. Measuring the longitudinal relaxation time of GABA in vivo at 3 Tesla. J Magn Reson Imaging. 2013;37(4):999–1003.

9. Mlynárik V, Gruber S, Moser E. Proton T1 and T2 relaxation times of human brain metabolites at 3 Tesla. NMR Biomed. 2001;14(5):325–31.

10. Choi C, Zhao C, Dimitrov I, Douglas D, Coupland NJ, Kalra S, et al. Measurement of Glutathione in Human Brain at 3T using an Improved Double Quantum Filter In Vivo. J Magn Reson. 2009;198(2):160–6.

11. Intrapiromkul J, Zhu H, Cheng Y, Barker PB, Edden RAE. Determining the in vivo transverse relaxation time of GABA in the human brain at 7T. J Magn Reson Imaging. 2013;38(5):1224–9.

12. An L, Li S, Shen J. Simultaneous determination of metabolite concentrations, T1 and T2 relaxation times. Magn Reson Med. 2017;78(6):2072–81.

13. Bartha R, Michaeli S, Merkle H, Adriany G, Andersen P, Chen W, et al. In vivo 1H2O T2† measurement in the human occipital lobe at 4T and 7T by Carr-Purcell MRI: Detection of microscopic susceptibility contrast. Magn Reson Med. 2002;47(4):742–50.

14. Wyss PO, Bianchini C, Scheidegger M, Giapitzakis IA, Hock A, Fuchs A, et al. In vivo estimation of transverse relaxation time constant (T2) of 17 human brain metabolites at 3T. Magn Reson Med. 2018;80(2):452–61.

15. Zhang X, Petersen ET, Ghariq E, De Vis JB, Webb AG, Teeuwisse WM, et al. In vivo blood T1 measurements at 1.5 T, 3 T, and 7 T. Magn Reson Med. 2013;70(4):1082–6.

16. Deelchand DK, Auerbach EJ, Kobayashi N, Marjańska M. Transverse relaxation time constants of the five major metabolites in human brain measured in vivo using LASER and PRESS at 3 T. Magn Reson Med. 2018;79(3):1260–5.

17. Posse S, Otazo R, Caprihan A, Bustillo J, Chen H, Henry PG, et al. Proton echo-planar spectroscopic imaging of J-coupled resonances in human brain at 3 and 4 Tesla. Magn Reson Med. 2007;58(2):236–44.

18. Mlynárik V, Gambarota G, Frenkel H, Gruetter R. Localized short-echo-time proton MR spectroscopy with full signal-intensity acquisition. Magn Reson Med. 2006;56(5):965–70.
